# The origins of Novo Nordisk and Novartis products: piloting a framework to identify the public contributions

**DOI:** 10.1080/20523211.2025.2534919

**Published:** 2025-08-05

**Authors:** Daniel Fabian, Ozren Sehic, Claudia Wild

**Affiliations:** Austrian Institute for Health Technology Assessment (AIHTA), Vienna, Austria

**Keywords:** Public contributions, Novo Nordisk, Novartis, product origin, public return on public investments (PRoPI), transparency, research and development (R&D), pharmaceuticals

## Abstract

**Background:**

The objective of this case study is to pilot a framework of public contributions examining the origins of products from two major European pharmaceutical companies, Novartis and Novo Nordisk, that received approval from the European Medicines Agency (EMA) between January 2014 and May 2024. Our primary focus was to investigate the extent of public contributions, including government grants, public-private partnerships, and other forms of public funding, that supported the development of these products. Additionally, we explored whether these companies primarily relied on in-house research and development (R&D) capabilities or acquired these products at various stages of their development.

**Methods:**

We conducted a thorough analysis of the products approved during the specified period, identifying the origins of each product. The analysis included detailed examination of public databases, financial disclosures, and scientific publications to trace the flow of public funding. We built on a list of sources from our previous studies to increase the level of detail.

**Results:**

Novartis demonstrated a tendency to acquire promising products and technologies from smaller biotech firms and other pharmaceutical companies, particularly in therapeutic areas where it sought to strengthen its market position like oncology (16 out of 25 products acquired, licensed or co-developed). Conversely, Novo Nordisk predominantly advanced its products through internal R&D efforts, although it also engaged in selective acquisitions to complement its core capabilities (two out of six products acquired, licensed or co-developed). For Novartis eleven products received public support, for Novo Nordisk one product did.

**Conclusion:**

Our findings reveal that both Novartis and Novo Nordisk use strategic acquisitions with Novartis relying more heavily on it than Novo Nordisk. Our framework for analyzing public contributions was sufficient for the product portfolios of the firms analyzed and helped us identifying public contributions.

## 1. Background

The pharmaceutical industry benefits substantially from public funding for research and development (R&D), yet transparency regarding these contributions remains limited. This challenge is compounded by the industry’s transformation through strategic mergers and acquisitions (M&A) to boost revenues and expand portfolios (Comanor & Scherer, 2013). As companies increasingly acquire external knowledge through patents and acquisitions rather than relying on in-house R&D (Jansen, 2008), publicly-funded innovations are often transferred to private companies, making it difficult to track the original public investment in drug development, a concern highly relevant for discussions about fair pricing and access to medicines.

Public institutions play a crucial role in pharmaceutical innovation, conducting basic research that directly benefits pharmaceutical firms while increasingly participating in later development stages. Research by Cleary et al. shows that every FDA-approved drug between 2000 and 2016 received some form of U.S. public health funding for R&D (Galkina Cleary et al., 2018) while only 20% of CAR T cell trials are sponsored by the pharmaceutical industry (Hartmann et al., 2017). This pattern has been characterised as ‘the public pays twice’ and ‘risks are socialized, rewards privatized’ (Vogler et al., 2023), as taxpayers fund initial research and then pay again for resulting medicines. Despite extensive documentation, relevant policies remain underdeveloped.

The European Union’s revised pharmaceutical legislation proposal (April 2023) includes a new Directive (European Commission (EC), 2023) mandating transparency in public R&D support. Article 57 requires market authorisation applicants to disclose direct public funding for medicinal product development, covering pre-clinical and clinical stages but excluding indirect support like tax incentives or product un-specific basic research.

The intersection of public funding and private acquisition creates transparency challenges. Knowledge from publicly funded institutions is often sold to larger pharmaceutical companies (Wild et al., 2025). Recent research demonstrates that NIH investment in drug development is comparable to or exceeds industry investment when accounting for basic research, clinical failures, and cost of capital (Cleary et al., 2023). Gilead Sciences’ $11.9 billion acquisition of Kite Pharma (Gilead, 2017) exemplifies this: CAR T cell therapy initially developed with NCI and NIH grants totalling $204,288,340 (Singhroy, 2017) was transferred to private hands, obscuring the original public investment.

High-profile mergers like Bristol-Myers Squibb/Celgene (2022) and AbbVie/Allergan (2020) demonstrate how companies strengthen market positions through M&A (AbbVie, 2020; Parrish, 2022). Leading firms including Pfizer, Roche, and Johnson & Johnson sustain dominance through strategic acquisitions while divesting non-core assets. Gagnon and Volesky (2017) identified 293 such transactions among 13 major firms, criticising this as fostering a ‘quasi-cartel’ environment (Gagnon & Volesky, 2017).

This research pilots a framework to increase transparency by assessing public contributions (Wild et al., 2025; Wild & Fabian, 2023) to Europe’s fastest-growing pharmaceutical companies, Novo Nordisk and Novartis (Dunleavy, 2024). We analyze their EMA-approved products from the last decade (January 2014–May 2024) to determine whether these companies focused on in-house development or M&A. This analysis reveals how fast-growing companies’ expansion strategies may rely on acquiring publicly-funded innovations, highlighting the need for transparency mechanisms that track public investments in the pharmaceutical sector.

## 2. Methods

This research was inspired by a study by Jung et al. on two US-based companies (Pfizer and J&J) and the origins of their product portfolios (Jung et al., 2019). We built on a search strategy we used in previous research (Schmidt & Wild, 2020; Schmidt et al., 2021, 2022, 2024) and adapted it to find information about the products of Novo Nordisk and Novartis.

### 2.1. Selection of companies and products to analyze

Since Novo Nordisk and Novartis represent the fastest-growing and two of the largest pharmaceutical companies in Europe in 2023, we selected those two European companies as case studies for our analysis. Novo Nordisk has the biggest year-over-year sales increase of any big pharmaceutical company of 31% in 2023 (Dunleavy, 2024). Novartis increased its revenue despite spinning out Sandoz by 7.7% from 2022 to 2023 (Dunleavy, 2024). Therefore, we analyzed all the products these two companies submitted for approval and were authorised between 2014 and 2024.

### 2.2. Identification of generic or nonproprietary and proprietary designations

We started the research by compiling a list of all Novo Nordisk and Novartis products that received EMA approval within the last ten years, from January 2014 to May 2024 using data from EMA’s excel sheet on all medicines. From the excel sheet we extracted product name, approval status, therapeutic area, therapeutic agent, data of approval, orphan status and PRIME: priority medicines status. We limited the search to the parent companies themselves and excluded their subsidiaries. We then thoroughly searched for product identifiers, including initial numbers and character combinations, generic or nonproprietary names of active ingredients, and trade or brand names usually given at a later stage of development. This search was conducted using AdisInsight. This ensures that we start the product search as early as possible of its history before the final company gives the product a final brand name for marketing’s sake.

### 2.3. Identification of originator and of public contributions to the development

Next, we searched for the earliest reference of these generic or nonproprietary names in publications to identify the origin of the products. Medline was searched to identify early basic R&D support, and the corresponding publications were searched for affiliations with academic institutions and research grants mentioned. Then, databases on clinical trials (https://clinicaltrials.gov/, https://eudract.ema.europa.eu/) and on supranational institutions for research funding (https://cordis.europa.eu/; https://reporter.nih.gov/) were searched. Sponsor details, type of funding and amounts were extracted.

**Figure 1.**
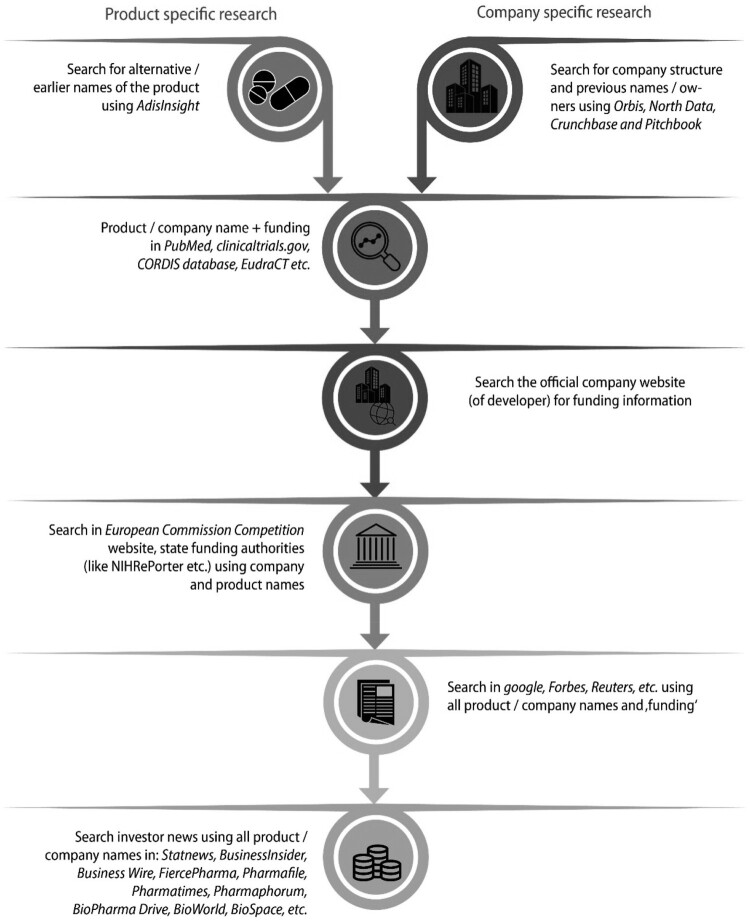
Search strategy for product/company information.

Finally, the official websites of Novartis and Novo Nordisk to find additional information, followed by searching the European Commission Competition website (https://competition-cases.ec.europa.eu/search) to determine whether any EU member states provided funds to the two companies, especially for developing the products in question. Figure 1 shows the steps taken to identify public support.

As a next and last step, we used Google, Forbes, Reuters to identify news articles about the products and finished the search using various investor news sources (Statnews, BusinessInsider, Business Wire, FiercePharma, Pharmafile, Pharmatimes, Pharmaphorum, BioPharma Drive, BioWorld, Biospace etc.). If the products or the knowledge that led to a product were acquired through an M&A, we also analyzed the originator company if they received public contributions. For detailed information on the stages of public contribution, category of public contributions, the funding agencies and sources of information see Table 1.

**Table 1.** Framework for analyzing public contributions to R&D of medical innovations applied on antibiotics R&D (Schmidt et al., 2024).

Public contribution by phase	Category of public contributions	Public & philanthropic sponsors (examples)	Sources	
Basic, applied & translational research	Basic, applied & translational research support (in form of R&D grants, public-private partnerships, basic knowledge creation by publicly funded research institutes etc.) Horizontal pre-competitive research support	National research funder: NIH, APC Microbiome Ireland, US Department of Defense, NIAID. Charity: Gates Foundation Public-private partnership: Health Holland, AMR Action Fund Regional/Local: St Vincent’s Hospital Melbourne, RMIT University	Europe: Cordis Db, IMI/IHI projects, Websites of National research agencies. US: NIH, US government agencies. Websites of other national research agencies	Following sources provide information on all categories: STATnews, FiercePharma, Biopharmadive, Pharmaphorum, Pharmatime, Pharmafile, Business Wire, BusinessInsider, BioPharma Drive, BioWorld, Biospace PRNewswire, Forbes, Reuters, … . Product + News (Google), Homepages (Developer): News, Press releases (ad-hoc announcements + press releases) Patents: FDA orange book/ US PTO, Health Canada Patent Database, Espacenet patent database, Medicines Patent Pool Patent, Pat-informed database
Early-stage research in SME and Biotech start-ups	Technology transfer grants for spinout/-off companies, Technology Business support for Lifesciences, biotech start-ups and SME	National research funder: National Science Foundation, UKRI, Innovate UK, Department of Defense, NIH, NIAID, SBIR, A*Star, BARDA, Supranational research funder: CARB-X, UK-China AMR Fund, IMI, EUHorizon 2020, EU Framework 7 National public investor: BpiFrance Supranational public investor: European Investment Bank Charity: CFF, Gates Foundation	Europe: EIC, EISMEA, EIT. US: SBIR/STTR seed funding. Google searches on Websites of Universities Websites of national support agencies e.g. Innovate UK, A*Star	
Late-stage development in Corporate Companies	Late-stage development in clinical trials Changes in ownership: licensing, acquisitions, merging	National research funder: FMRI, US Department of Defense, US Army Medical R&D Command, US Defense Health Agency, US Navy, US Defense Threat Reduction Agency, BARDA, NIH, NIAID, SBIR, UK Innovation Agency, INSERM, Chinese Academy of Medical Sciences, AIIMS, Australian Research Council, Australian Government. Supranational research funder: CARB-X, European & Developing Countries Clinical Trials Partnership, IMI Public-private Partnership: health Holland, AMR Action Fund Charity: Gates Foundation, CFF Regional: Imperial College, FISEVI	International: clinicaltrials.gov, ICTRP Europe: EudraCT/ CTIS Regional trial registries US: Securities & Exchange Commission (SEC-Reports) FDA/EMA submissions	
Market authorisation, Post Launch Evidence Generation	Regulatory support: Scientific Advice, fast track etc., Real-World-Evidence data collections & Post Launch Evidence Generation, tax credits	None found for this research found	Europe: EMA, HTA CG – SG JSC, IMI/IHI projects US: US Securities & Exchange Commission (SEC-Reports), FDA	

### 2.4. Application of framework on public funding

Finally, the framework for public contributions to R&D in medical innovations, as developed in previous project phases (Schmidt & Wild, 2020; Schmidt et al., 2021, 2022, 2024; Wild & Fabian, 2023; Wild et al., 2025), was used to classify the public contributions identified in the development of the products. These contributions can be of financial, organisational or intellectual etc. nature and were categorised by the phase of development in which they occurred and by their type (Table 1). The piloting of the framework was conducted in August 2024.

### 2.5. Identification of trends between public support and therapeutic value assessment

Drawing on therapeutic value assessments (measured with the ASMR rating) from the French HTA body HAS, we examined whether products developed in-house versus those with public contributions showed distinct patterns in their ASMR ratings (1 = major benefit, 5 = no benefit). We used the most recent ASMR ratings and used the ratings for all available indications (as of 3rd of July 2025).

## 3. Results

### 3.1. Novartis product approvals and public contributions to the product portfolio (2014–2024)

Between January of 2014 and May 2024, Novartis had 25 new products approved. Twelve products were indicated for oncological diseases (Adakveo®, Kisqali®, Kymriah®, Locametz®, Lutathera®, Mekinist®, Piqray®, Pluvicto®, Rydapt®, Scemblix®, Tabrecta®, Zykadia®), four for ophthalmologic diseases (Beovu®, Izba®, Luxturna®, Simbrinza®), three for neurological diseases(Aimovig®, Kesimpta®, Mayzent®), two for immunology and dermatology specialties (Cosentyx®, Lamisil®), two for cardiovascular diseases (Entresto®, Leqvio®), one for a hematology condition and oncological disease (Farydak®), and one for a neuromuscular disease (Zolgensma®).

**Figure 2.**
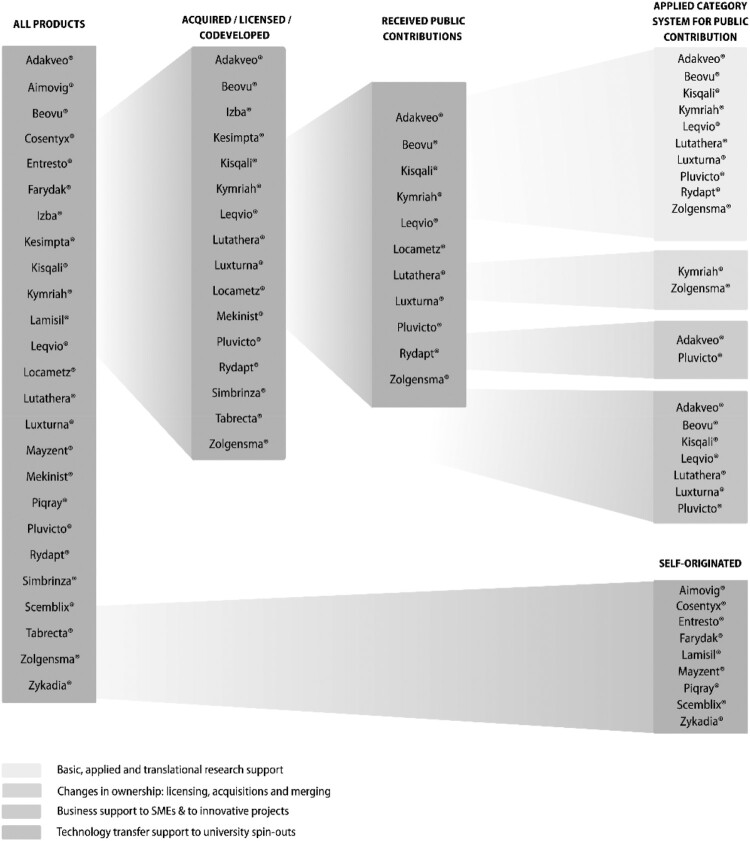
Novartis’ products that received EMA approval between January 2014 and May 2024.

16 of 25 products were acquired, licensed, or co-developed, representing 64% of their total portfolio approved in this period (see Figure 2). Novartis acquired six products from academic spin-outs: Adakveo®, Beovu®, Kisqali®, Luthatera®, Luxturna®, and Pluvicto®. One product (Leqvio®) was acquired from a research institute mostly financed by the public (Max-Plank Gesellschaft financed by the German state and regions). Two products were co-developed with publicly funded hospitals or universities (Rydapt®, Locametz®). The exclusive licensing right was bought for one product (Kymirah®) from the University of Pennsylvania. Another product (Zolgensma®) was licensed by AveXis (acquired by Novartis in May 2018) from the National Children’s Hospital, the University of Pennsylvania and ReGenX Biosciences (for detailed information, see Supplemental Material S1). The other nine products (Aimovig®, Cosentyx®, Entresto®, Farydak®, Lamisil®, Mayzent®, Piqray®, Scemblix® and Zykadia®) are self-originated.

Of the 25 products Novartis approved in 2014–2015, eleven (44%) benefited from public support through government grants, academic collaborations, or public institutions. When including products that were acquired, licensed-in, or co-developed, this figure increases to approximately 69%.

Among these the 25 products, eleven were supported by public contributions or were based on research funded by public sources, meaning 44% of Novartis’ products approved in 2014–2014 benefited from public support (e.g. government grants, academic collaborations, public institutions, etc.) and when also including products that were acquired, licensed-in, or co-developed, this percentage rises to roughly 69%.

When applying the framework for structured analysis of public contributions, we found that the public funds can be attributed to the categories: ‘Basic, applied and translational research support’ (ten products – 91% of publicly supported products), ‘Technology transfer support to university spin-outs’ (seven products – 64%), ‘Business support to SMEs & to innovative projects’ (two products – 18%) and ‘Changes in ownership: licensing, acquisitions and merging’ (two products – 18%), meaning that the public contributions is higher in the earlier stages of R&D and decrease until market authorisation (‘Basic, applied & translational research’: ten products – 91%; ‘Early stage research in SME and Biotech start-ups’: nine products – 82%, ‘Late stage development in Corporate Companies’: two products – 8%). For the category ‘Market Authorization, Post Launch Evidence Generation’ no information is available (for details see Table 2). Seven products of Novartis hold an orphan designation (Farydak®, Kymriah®, Lutathera®, Luxturna®, Rydapt®, Scemblix®, Zolgensma®) and of them five have received public support (Kymriah®, Lutathera®, Luxturna®, Rydapt®, Zolgensma®). Two products (Kymriah®, Zolgensma®) have a *PRIME: priority medicines designation*, meaning they receive certain advantages such as early, proactive and enhanced support to medicine developers (European Medicines Agency (EMA), 2024b).

**Table 2.** Products of Novartis that received public contributions and the applied category system.

Product name	Medical specialty	Category and type of public contribution	Details on public contribution
Adakveo®	Oncology	Business support to SMEs & to innovative projects Basic, applied and translational research support Technology transfer support to university spin-outs.	National funding: 5 grants awarded from the Small Business Innovative Research (SBIR) of in total 12.427.859 USD from 2004 to 2012. National funding: Selexys Pharmaceuticals which is a University of Oklahoma spin-out was acquired by Novartis in 2016.
Beovu®	Ophthalmology	Basic, applied and translational research support Technology transfer support to university spin-outs	National funding: University of Zürich spin-out.
Kisqali®	Oncology	Basic, applied and translational research support Technology transfer support to university spin-out	National funding: University of Cambridge spin-out
Kymriah®	Oncology	Basic, applied and translational research support Changes in ownership: licensing, acquisitions and merging University of Pennsylvania	National funding: Developed by University of Pennsylvania and sold exclusive licensing to Novartis in 2012.
Leqvio®	Cardiology	Basic, applied and translational research support Technology transfer support to university spin-out	National funding: Novartis acquired ‘The Medicines company’ which is a spin-out of the Max Planck Gesellschaft. (mostly German state financed organisation)
Locametz®	Oncology	Basic, applied and translational research support	National funding: Ga 68 PSMA-11 (alternative name) was co-developed by researchers at University of California, Los Angeles and University of California, San Francisco, who conducted a phase III clinical trial with Advance Accelerator Applications (AAA) which was acquired by Novartis in 2018.
Lutathera®	Oncology	Basic, applied and translational research support Technology transfer support to university spin-outs	National funding: Startup researchers developed the drug while being paid by University of Rotterdam. National funding: Phase 1 trial was carried out in Erasmus MC. Supranational and national funding: Acquired by AAA. AAA was acquired by Novartis for 3.9 billion USD in 2018.AAA is a spin-out from the European Organization for Nuclear Research (CERN).
Luxturna®	Ophthalmology	Basic, applied and translational research support Technology transfer support to university spin-outs	National funding: Spark Therapeutics is a spin-out from and Children’s Hospital of Philadelphia which co-developed the drug with the University of Pennsylvania. Roche bought Spark Therapeutics and holds the rights to the drug in the US, while Novartis holds the rights outside the US.
Pluvicto®	Oncology	Basic, applied and translational research support Technology transfer support to university spin-outs Business support to SMEs & to innovative projects	National funding: Endocythe is a university spin-out of Purdue University (public universiry) Regional funding: In 2001 Endocyte received 2 million USD from Indiana’s twenty-first Century Research and Technology Fund to continue clinical development of its oncology diagnostic and therapeutic products, purchase additional research equipment, and increase its research and development staff. National funding: Phase II clinical trial conducted by the Australian and New Zealand Urogenital and Prostate Cancer Trials Group (ANZUP) and the University of Sydney which jointly have the primary financial obligation and Endocyte providing financial support in exchange for access to data.
Rydapt®	Oncology	Basic, applied and translational research support	Too little information to classify funders: Collaboration with different academic institutions (no further information on the academic institutions found).
Zolgensma®	Neuromuscular	Basic, applied and translational research support Changes in ownership: licensing, acquisitions and merging	National funding: The University of Pennsylvania has highlighted James Wilson’s pivotal role in the development of the technology. According to the NIH RePORTER, James Wilson received over 35.8 million USD in funding from the NIH for research related to ‘adeno-associated virus’ while at Penn. At NCH, researchers who worked on the development of Zolgensma included Brian Kaspar and Jerry Mendell, two scientists who have received more than 25 million USD in NIH grants, including millions for work on SMA. National funding: AveXis (which was acquired by Novartis) licensed patents related to Zolgensma from Nationwide Children’s Hospital (NCH), the University of Pennsylvania, REGENX Biosciences (a firm created in 2009 by the University of Pennsylvania), and Genethon (a French charity).

Information on funding amounts is available for only some products: Adakveo® (Business support to SME: roughly 12.4 million USD), Pluvicto® (Business support to SME: 2 million USD) and Zolgensma® (Basic research support: 35.8 million USD and 25 million USD).

No direct public contributions could be identified for eleven products (Aimovig®, Cosentyx®, Entresto®, Farydak®, Izba®, Kesimpta®, Lamisil®, Mayzent®, Mekinist®, Piqray®, Scemblix®, Simbrinza®, Tabrecta®, Zykadia®)

### 3.2. Novo Nordisk Product approvals and public contributions to the product portfolio (2014–2024)

**Figure 3.**
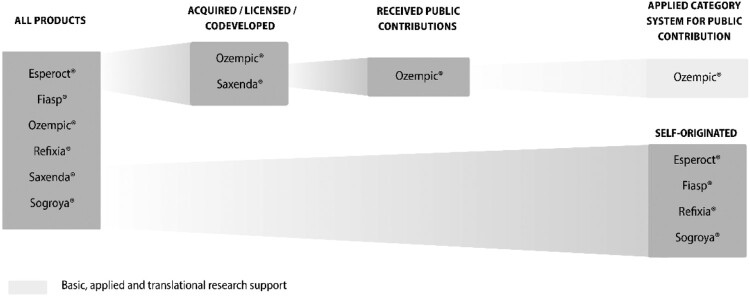
Novo Nordisk’ products that received EMA approval between January 2014 and May 2024.

Novo Nordisk had six (see Figure 3) new products approved in this time period. Four products were indicated for endocrine diseases (Fiasp®, Ozempic®, Saxenda®, Sogroya®) and two for hematological diseases (Esperoct®, Refixia®).

For Novo Nordisk one product (Saxenda®) was co-developed with the University of Copenhagen. Ozempic® is based on research conducted by various academic researchers, raising questions about the public contribution to the development of this product (for detailed information see Supplemental Material S2). The remaining four products (Esperoct®, Fiasp®, Refixia®, Sogroya®) are self-originated.

Two out of six or 33% of Novo Nordisk’s new products were either acquired, licensed, or co-developed (Saxenda®, Ozempic®). Ozempic® is the only new product of Novo Nordisk that received public contributions of some sorts (see Supplemental Material S2 for detailed information), meaning one out of six products received public contributions (16.6%). Sogroya® is the only new product of Novo Nordisk that has an orphan designation meaning it received benefits such as protocol assistance, access to centralised authorisation procedure, ten years of market exclusivity and/or fee reductions (European Medicines Agency (EMA), 2024a).

When applying the framework for structured analysis of public contributions, we found that the only category Novo Nordisk was publicly supported was in ‘Basic, applied and translational research support’ (see Table 3). No further public funding and no detailed information on amounts could be identified.

**Table 3.** Products of Novo Nordisk that received public contributions and the applied category system.

Product name	Medical specialty	Category and type of public contribution	Details on public contribution
Ozempic®	Endocrinology	Basic, applied and translational research support	Controversy based on publically funded research on GLP-1 (alternative name). National funding: Researchers Jens Juul Holst and Joel Habener received funding from Novo Nordisk on their GLP-1 research but did not patent their research on GLP-1. Basic research from Svetlana Mojsov played a critical role in the development on GLP-1 but she was not directly involved with the development of Semaglutide (therapeutic agent).

No direct public contributions could be identified for the remaining five products (Esperoct®, Fiasp®, Refixia®, Saxenda®, Sogroya®).

### 3.3. Assessment of added therapeutic value

The data reveals patterns in how public funding and acquisition strategies correlate with therapeutic innovation as measured by French ASMR ratings. Of the 31 products in total, we found ASMR ratings for 25 of them (as seen in Supplemental Material S1 and S2).

**Figure 4.**
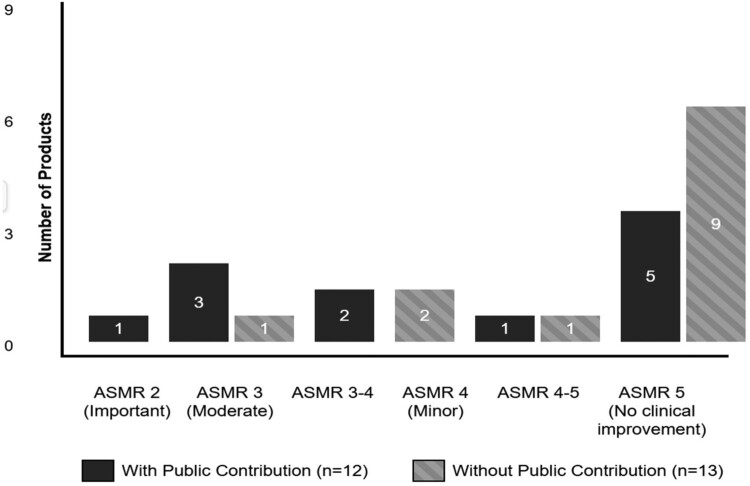
Distribution of Therapeutic Value Ratings (ASMR) by public funding status.

Products with documented public contributions demonstrate better ASMR ratings (see Figure 4). Among the 12 products with public support, 50% achieved ASMR ratings of 2–3 (important to moderate improvement), including all three gene/cell therapies: Luxturna® (ASMR 2), Zolgensma® (ASMR 3), and Kymriah® (ASMR 3-4). In contrast, products without identified public contributions predominantly received ASMR 5 ratings (69%), indicating no therapeutic improvement over existing treatments.

**Figure 5.**
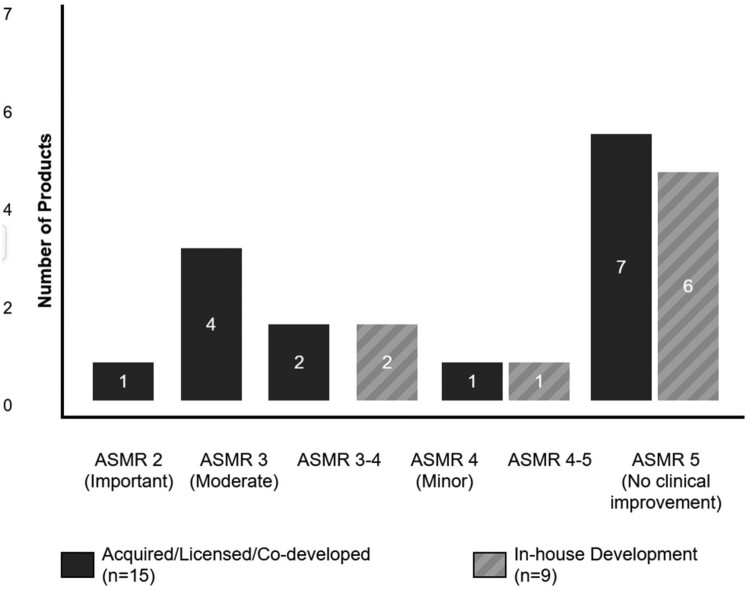
Distribution of Therapeutic Value Ratings (ASMR) by development origin.

The relationship between development origin and therapeutic value is equally compelling. Acquired or licensed products show a clear tendency toward higher therapeutic value, with 46% achieving ASMR ratings of 2–3 (see Figure 5). Notably, the single product achieving ASMR 2 (Luxturna®) was acquired from an academic spin-out. In stark contrast, in-house developed products cluster heavily at the lower end of the scale, with 67% receiving ASMR 5 ratings and none achieving better than ASMR 4.

## 4. Discussion

To summarise, Novartis had a significantly higher number of recently approved (from 2014 to 2024) products compared to Novo Nordisk (25 vs. 6). Also, a more significant proportion of Novartis’ products were either acquired, licensed, or co-developed (64% vs. 33%). Additionally, a higher percentage of Novartis’ products benefited from public contributions (44% of their total vs. 16.6% for Novo Nordisk). In total, we found public contributions to 16 of 31 drug developments analyzed. 14 public contributions came from national funding, one from supranational and one from regional funders. This analysis underscores the significant role that public funding plays in developing new drugs, particularly in the early stages of product development. This finding suggests that even among Europe’s most commercially successful pharmaceutical companies, public sector investments remain integral to filling drug development pipelines.

Our findings reveal diverse approaches to building product portfolios in the European pharmaceutical industry. While some products emerge from internal R&D programmes, a substantial portion (52% across both companies) involved acquisitions, licensing, or co-development arrangements. This suggests that highly successful European pharmaceutical companies rely significantly on external innovation sources, often originating from publicly funded institutions. The prevalence of university spin-outs and academic collaborations among acquired products underscores the critical role of technology transfer from public to private sectors.

The application of the public support framework proved to work well and allowed for the classification of both the stage of product development and the nature of public contributions. Utilising this framework facilitated the structured approach to compiling diverse information from different funding sources, enabling a systematic analysis of the type of funding. Piloting the framework on products from two large pharmaceutical companies showed us that early-stage research support is strongly represented among the products. At the same time, later stages of development were less represented. Public support for basic research was frequent for oncological products in particular. However, information on ‘marketing authorization, post-launch evidence generation’ was missing for all of the medicinal products examined. No documented data could be determined, as this data is difficult to collect, even though managed entry agreements are now common, albeit opaque. Therefore, further research needs to be conducted on the framework to include more sources that capture such public support.

In the findings from our case study on the product portfolios of Novartis and Novo Nordisk, we found that the amount of self-originated products is much greater than what we expected when compared to the study that inspired this research on Pfizer and Johnson & Johnson (J&J) (Jung et al., 2019). Nevertheless, our results cannot be directly compared to the results of the study by Jung et al. since the methodology varies greatly (the authors did not specify the exact sources but said they ‘gathered information on the discovery and early development of these products from peer-reviewed publications, media reports, and company press releases’ [Jung et al., 2019]). Therefore, assumptions about the ‘innovativeness’ of these four companies (Novartis, Novo Nordisk, Pfizer, J&J) cannot be made; rather, the complementarity of the public and private sectors is stressed. Furthermore, we cannot guarantee that we found all public contributions – eventually, more products may have received public contributions but were not identified by our search methodology.

Instead of primarily relying on M&A to ‘fill’ the pipelines, Novo Nordisk seems to develop more in-house products than Novartis. A recent analysis ranks Novo Nordisk above their peers in the annual Pharmaceutical Invention Index for 2024 (Herper, 2024), which further explains that the enormous increase in the revenues and the success of Ozempic® and Wegovy® led Novo Nordisk to continue their research in weight loss medications with many phase II and phase III trials currently being conducted (Pharma, 2024). This difference highlights a broader reliance on external publicly supported innovation of Novartis’ portfolio compared to Novo Nordisk, where in-house development plays a more prominent role.

However, comparing Novartis and Novo Nordisk seems reasonable only on the surface. We must also take the focus of their development programmes into account. Novartis targets a wide range of diseases, especially in oncology, and Novo Nordisk has a strong focus on diabetes. Cancer is not a single disease but many distinct diseases, each with genetic, molecular, and external characteristics. This heterogeneity makes the development of treatments particularly challenging, as therapies must be tailored to specific cancer types, stages, and even individual genetic profiles. Cancer cells can rapidly evolve and resist treatments, necessitating the continuous development of new drugs or combination therapies. This resistance complicates both the treatment strategy and the clinical trial design (Fouad & Aanei, 2017; Hanahan & Weinberg, 2011). Diabetes, particularly type 2, is more homogeneous in its presentation, primarily involving insulin resistance and/or insulin deficiency. This makes the disease somewhat more predictable in terms of treatment development. There is a more extended history of effective treatment options for diabetes, such as insulin, metformin, and newer classes like GLP-1 receptor agonists (Karamanou et al., 2016). This existing knowledge base provides a foundation that can be built upon for new treatments.

The R&D of oncology products are generally more challenging than those of diabetes treatments due to the complexity, variability, and aggressive nature of cancer. While diabetes R&D is also challenging, it is more predictable and less prone to high failure rates as in oncology. This could be why Novartis has a higher number of products that were not developed internally but acquired to fill up the R&D pipeline.

When analyzing the therapeutic value ratings with development origin and public contributions, we can conclude that the most innovative products share three characteristics: they originate from academic institutions, receive substantial public funding, and are subsequently acquired by pharmaceutical companies. This pattern is exemplified by the gene and cell therapies that achieved the highest ASMR ratings. Conversely, traditional in-house pharmaceutical R&D appears to produce primarily incremental innovations, with most products offering no therapeutic advantage over existing treatments. When putting our results in context with previous works analyzing public contributions in context of therapeutic novelty or added-benefit, our results are in line with the wider literature (Galkina Cleary et al., 2018; Ledley & Cleary, 2023; Sampat & Lichtenberg, 2011). Public contributions tend to go towards therapeutic fields with worse treatment options instead of ‘me-toos’.

We found that searching in investor news is the most helpful place to find information about product origins and funding. M&A, significant funding that the companies receive, exclusive licensing, and promising clinical trial results are all frequently posted on the included websites, which makes searching in them the easiest and most rewarding. In contrast, searching the European Commission Competition website was the least fruitful source for funding information. If the companies received funding, it was usually for promoting economic activity by supporting local factories or research institutes but not for continuing specific research. Therefore, such public contributions cannot be attributed to any specific products.

### 4.1. Limitations

A key limitation is our selection of case studies based solely on rapid growth rates, which may not yield representative findings for the broader pharmaceutical industry. Novo Nordisk’s 31% growth is particularly idiosyncratic, driven largely by Ozempic’s success in obesity treatment rather than typical R&D patterns. Alternative selection criteria – such as company size, therapeutic focus, or contrasting small versus large firms – might have provided more general insights into public contributions to pharmaceutical innovation. The substantial differences between our two highlight how company specific factors limit the broader applicability of our findings.

A significant limitation of this research is the scope of sources utilised. Although a comprehensive array of sources was consulted, including peer-reviewed journals, industry reports, investor news, and regulatory documents, the breadth of the pharmaceutical industry means that it is difficult to encompass every relevant piece of literature. Future studies would benefit from working with our framework and list of sources and extending the range, potentially including more grey literature, patents, and non-English publications. We cannot claim to provide an exhaustive list of sources of financial support or supporting institutions.

Most of the sources in this study were in English and German, which may have excluded valuable insights and data from research conducted in other languages. The linguistic limitations inherent in this study may have inadvertently restricted the comprehensiveness of the research. The predominance of English and German sources means that significant R&D published in other languages could have been overlooked. This is particularly relevant in the context of global pharmaceutical research, where countries like China, Japan, and France contribute extensively to scientific literature. Incorporating non-English sources in future research could provide a more detailed understanding of the origins of knowledge in the pharmaceutical industry.

Jurisdictional variations in pharmaceutical regulations and funding transparency create significant limitations, as countries range from stringent disclosure requirements for clinical trials and adverse effects to lenient policies allowing companies to withhold information. Similarly, funding sources carry different transparency obligations – public funding typically mandates detailed financial reporting while private funding often requires less disclosure. These disparities across regions and funding types result in an incomplete industry picture and make it challenging to fully assess the impact of funding sources on pharmaceutical R&D.

These limitations outlined above may produce an incomplete or skewed understanding of pharmaceutical practices, leaving aspects of less transparent regimes underexplored. Future research should broaden linguistic scope beyond European sources for a global perspective, conduct comparative analyses of legislative environments’ impact on transparency, and include diverse funding sources (non-EU and North American) to comprehensively evaluate their influence on outcomes, thereby providing a more robust understanding of public contributions to drug development.

## 5. Conclusion

The pharmaceutical industry is undergoing profound changes, characterised by the emergence of large conglomerates through M&A. The sector benefits from a wealth of knowledge created with public funds and experience, scientific infrastructure, global economic growth, low cost of borrowing money, increased societal knowledge, and a growing, highly educated labour force. Our case study on Novartis and Novo Nordisk showed that a substantial number of their products that received EMA approval in the last 10 years were not in-house developments and have received substantial public contributions (in the forms of grants or basic knowledge being developed with public funding in public universities). Furthermore, we found that novel therapeutic products that provide a benefit to existing systems often are publicly funded. Therefore, we can conclude that M&A is important in ‘filling’ the product pipelines. However, the magnitude of public contributions varies greatly for companies with different specialisations.

Among the total number of products from two large European pharmaceutical companies (Novartis and Novo Nordisk) approved within the last ten years, many have received public contributions. When applying the framework for a structured analysis of these public contributions, we found that public contributions are more prominent within the early stages of drug development (in basic and applied research, in technology transfer support to university spin-outs and business support to SMEs) than in later stages. Data for public contributions to market authorisation (discounts for orphan drugs) and to post-launch evidence generation (data collections for managed-entry agreements) was hardly found. Various reasons are plausible, the explanation being the difficulty of capturing these categories with the methodology used. Focusing primarily on affiliations of researchers in the early stages, company websites, funding agencies and investor news, indirect benefits are largely not captured. When applying our methodology, we found a need to expand the sources further.

## CRediT authorship contribution statement

Daniel Fabian: Conceptualization; Data curation; Formal analysis; Investigation; Methodology; Project administration; Validation; Visualization; Writing – Original Draft and Review & Editing Claudia Wild: Conceptualization; Formal analysis; Methodology; Project administration; Supervision; Validation; Writing – review & editing. Sehic Ozren: Methodology; Data curation. This work was conducted with the assistance of AI (Claude version Opus 4) for increasing readability and concise language use

## Supplementary Material

Supplemental Material
