# Supplementary material for: The origins of Novo Nordisk and Novartis products: piloting a framework to identify the public contributions
Source: J Pharm Policy Pract. 2025 Aug 5;18(1):2534919. doi: 10.1080/20523211.2025.2534919 (PMC12326385; doi:10.1080/20523211.2025.2534919)
Supplement: Supplemental Material [file JPPP_A_2534919_SM7769.docx]

Supplemental Material

Supplement 1 Novartis' products that received EMA approval between 2014 and May 2024

| Origin company | Information on acquisitions | Public contribution | Type and categories of public funding | Source |
| --- | --- | --- | --- | --- |
| Adakveo®  *Therapeutic agent: Crizanlizumab*  *Alternative names: Crizanlizumab-tmca - Novartis; SEG-101; Sel G1*  *Medical specialty: Oncology*  *Pharmacotherapeutic group: Other hematological agents*  *Therapeutic area: Anemia, Sickle Cell*  *Orphan medicine: No*  *PRIME: priority medicines: No*  *Date of approval: 28.10.2020 (authorization has been revoked in 2023)*  *ASMR rating: 5* | | | | |
| Selexys Pharmaceuticals | Novartis originally scored the drug through the 665 million USD Selexys Pharmaceuticals buyout in 2016. | YES | Business support to SMEs & to innovative projects:  5 grants awarded from the Small Business Innovative Research (SBIR) of in total 12.427.859 USD from 2004 to 2012  Basic, applied and translational research support and  Technology transfer support to university spin-outs:  University of Oklahoma | https://shorturl.at/5EoZ7  https://shorturl.at/AkTnH |
| Aimovig®  *Therapeutic agent: Erenumab*  *Alternative names: AMG-334; Erenumab-aooe*  *Medical specialty: Neurology*  *Pharmacotherapeutic group: Analgesics*  *Therapeutic area: Migraine Disorders*  *Orphan medicine: No*  *PRIME: priority medicines: No*  *Date of approval: 26.07.2018*  *ASMR rating: 5* | | | | |
| Codeveloped by Amgen and Novartis | n.a. | n.a. | n.a. | https://shorturl.at/T6kEu |
| Beovu®  *Therapeutic agent: Brolucizumab*  *Alternative names: Beobyu; Brolucizumab-dbll; ESBA-1008; RTH-258*  *Medical specialty:* *Ophthalmology*  *Pharmacotherapeutic group: Ophthalmologicals*  *Therapeutic area: Wet Macular Degeneration*  *Orphan medicine: No*  *PRIME: priority medicines: No*  *Date of approval: 13.02.2020*  *ASMR rating: 5* | | | | |
| EsbaTech | Spin-Out of University of Zurich (1998), taken over by Alcon, fully acquired by Novartis in 2010. | YES | Basic, applied and translational research support and  Technology transfer support to university spin-outs:  University of Zürich | <https://shorturl.at/z5m3x> |
| Cosentyx®  *Therapeutic agent: Secukinumab*  *Alternative names: AIN-457; Anti-interleukin 17A monoclonal antibody; KB-03303A; Kosentikusu; NVP-AIN-457; Scapho*  *Medical specialty:* *Immunology and Dermatology*  *Pharmacotherapeutic group: Immunosuppressants*  *Therapeutic area: Arthritis, Psoriatic;Psoriasis;Spondylitis, Ankylosing*  *Orphan medicine: No*  *PRIME: priority medicines: No*  *Date of approval: 14.01.2015*  *ASMR rating: 4-5* | | | | |
| Alcon; Novartis | Acquisition in 2010 followed by a company spin-out of Novartis in 2019. | n.a. | n.a. | <https://shorturl.at/PFhPu> |
| Entresto®  *Therapeutic agent: sacubitril, valsartan*  *Alternative names: AHU-377; dapagliflozin; Enrest; Enresuto; ENTRESTO SPRINKLE; LCZ-696; LCZ-696A; Neparvis; Valsartan/AHU-377*  *Medical specialty:* *Cardiovascular, Renal and Metabolism*  *Pharmacotherapeutic group: Angiotensin II antagonists, other combinations Agents acting on the renin-angiotensin system*  *Therapeutic area:* *Heart Failure*  *Orphan medicine: No*  *PRIME: priority medicines: No*  *Date of approval: 19.11.2015*  *ASMR rating: 4* | | | | |
| Novartis | n.a. | n.a. | n.a. | n.a. |
| Farydak®  *Therapeutic agent: Panobinostat*  *Alternative names: Faridak; LBH-589; LBH-589A*  *Medical specialty: Hematology, oncology*  *Pharmacotherapeutic group: Antineoplastic agents*  *Therapeutic area:* *Multiple Myeloma*  *Orphan medicine: Yes*  *PRIME: priority medicines: No*  *Date of approval: 20.02.2014*  *ASMR rating: 5* | | | | |
| Novartis | n.a. | n.a. | n.a. | n.a. |
| Izba®  *Therapeutic agent: Travoprost*  *Alternative names: AL 06221; AL-6221; Travatan; Travatan APS; Travatan Z; Travatanz; Travoprost advanced preservative system; Travoprost APS*  *Medical specialty:* *Ophthalmology*  *Pharmacotherapeutic group: Ophthalmologicals, Antiglaucoma preparations and miotics*  *Therapeutic area: Ocular Hypertension;Glaucoma, Open-Angle*  *Orphan medicine: No*  *PRIME: priority medicines: No*  *Date of approval: 28.08.2015*  *ASMR rating: 5* | | | | |
| Alcon | Acquisition in 2010 followed by a company spin-out of Novartis in 2019. | n.a. | n.a. | <https://shorturl.at/zBmAm> |
| Kesimpta®  *Therapeutic agent: Ofatumumab*  *Alternative names: Anti-CD20 monoclonal antibody - Genmab; Arzerra; GSK-1841157; HuMax CD20; OMB-157*  *Medical specialty:* *Neurology*  *Pharmacotherapeutic group: Immunosuppressant*  *Therapeutic area:* *Multiple Sclerosis, Relapsing-Remitting*  *Orphan medicine: No*  *PRIME: priority medicines: No*  *Date of approval: 26.03.2021*  *ASMR rating: 3* | | | | |
| Genmab | Kesimpta was originally developed by Genmab and licensed to GlaxoSmithKline; Novartis obtained rights for ofatumumab from GSK in all indications, including RMS, in December 2015. | n.a. | n.a. | <https://shorturl.at/cGLTF> |
| Kisqali®  *Therapeutic agent: Ribociclib*  *Alternative names: LEE-011; LEE-011A*  *Medical specialty:* *Oncology*  *Pharmacotherapeutic group: Antineoplastic agents*  *Therapeutic area: Breast Neoplasms*  *Orphan medicine: No*  *PRIME: priority medicines: No*  *Date of approval: 22.08.2017*  *ASMR rating: 3-4* | | | | |
| Astex Therapeutics (in collaboration with Novartis); Mount Sinai Health System | University of Cambridge spin-out (1999). | YES | Basic, applied and translational research support and  Technology transfer support to university spin-outs:  University of Cambridge | <https://shorturl.at/rrWuH> |
| Kymriah®  *Therapeutic agent:* *Tisagenlecleucel*  *Alternative names: Anti-CD19-CAR transduced T cells; Anti-CD19-chimeric-antigen-receptor-peripheral-blood-lymphocytes; Anti-CD19-chimeric-antigen-receptor-retroviral-vector-transduced-autologous-T-cells; Anti-CD19-chimeric-immune-receptor-retroviral-vector-transduced-autologous-T-cells-Novartis; Anti-CD19-CIR-retroviral-vector-transduced-autologous-T-cell-therapy; CART-019; CART-19; CART-19 cells; CART-19-cells; CD19-targeted-chimeric-antigen-receptor-immunotherapy; Chimeric antigen receptor-modified T cells against CD19; CTL-019; Kimria; LG-740; tisagenlecleucel; tisagenlecleucel-T*  *Medical specialty: Oncology*  *Pharmacotherapeutic group: Other antineoplastic agents*  *Therapeutic area: Precursor B-Cell Lymphoblastic Leukemia-Lymphoma;Lymphoma, Large B-Cell, Diffuse*  *Orphan medicine: Yes*  *PRIME: priority medicines: Yes*  *Date of approval: 22.08.2017*  *ASMR rating: 3-4* | | | | |
| Lentigen Corporation; University of Pennsylvania | Developed bv University of Pennsylvania and sold exclusive licensing to Novartis in 2012. | YES | Basic, applied and translational research support  Changes in ownership: licensing, acquisitions and merging:  University of Pennsylvania | <https://shorturl.at/7mfDr>  https://shorturl.at/c4tbX |
| Lamisil®  *Therapeutic agent:* *Terbinafine*  *Alternative names: DermGel;Ramicil; SDZ SF86327; SF 86327*  *Medical specialty:* *Dermatology*  *Pharmacotherapeutic group: Infectious diseases*  *Therapeutic area: pityriasis versicolor, fungal nail infections*  *Orphan medicine: No*  *PRIME: priority medicines: No*  *Date of approval: 22.08.2017*  *ASMR rating: 5* | | | | |
| Novartis | n.a. | n.a. | n.a. |  |
| Leqvio®  *Therapeutic agent:* *Inclisiran*  *Alternative names: ALN-60212; ALN-PCSsc; KJX-839;PCSK9si*  *Medical specialty: Cardiology*  *Pharmacotherapeutic group: Lipid modifying agents*  *Therapeutic area: Hypercholesterolemia;Dyslipidemias*  *Orphan medicine: No*  *PRIME: priority medicines: No*  *Date of approval: 09.12.2020*  *ASMR rating: 5* | | | | |
| Alnylam Pharmaceuticals | Novartis acquired The Medicines Company (which acquired the rights to ALN-PCS from Alnylam) for 9.7 bn USD in 2019 | YES | Basic, applied and translational research support and  Technology transfer support to university spin-outs:  The Medicines company is a spin-out of the Max Planck Gesellschaft  (mostly German state and regions financed organization) | <https://shorturl.at/4krjZ>  <https://shorturl.at/m3BuS>  <https://shorturl.at/Ipv2X> |
| Locametz®  *Therapeutic agent:* Gallium Ga 68 gozetotide  *Alternative names: Gozetotide; 68)Ga labeled Glu-NH-CO-NH-Lys(Ahx)-HBED-CC; (68)Ga-labeled Glu-urea-Lys(Ahx)-HBED-CC; (68)Ga-PSMA Ligand Glu-urea-Lys(Ahx)-HBED-CC; (68)Gallium-PSMA Ligand Glu-urea-Lys(Ahx)-HBED-CC; (68Ga)Glu-urea-Lys(Ahx)-HBED-CC; (68Ga)PSMA-HBED-CC; 68Ga-DKFZ-PSMA-11; 68Ga-HBED-CC-PSMA; 68Ga-HBED-PSMA; 68Ga-labeled Glu-NH-CO-NH-Lys(Ahx)-HBED-CC; 68Ga-PSMA; 68Ga-PSMA HBED-PET/CT; 68Ga-PSMA ligand Glu-urea-Lys(Ahx)-HBED-CC; 68Ga-PSMA-11; 68Ga-PSMA-HBED-CC; 68Ga-PSMA-ligand; [68Ga] Prostate-specific Membrane Antigen 11; [68Ga]GaPSMA-11; Ga PSMA; Ga-68 labeled DKFZ-PSMA-11; Ga-68 labeled Glu-NH-CO-NH-Lys(Ahx)-HBED-CC; Ga-68 labeled Glu-urea-Lys(Ahx)-HBED-CC; Ga-68 labeled PSMA-11; Ga-68 labeled PSMA-11 PET; Ga-68-PSMA-11; Gallium Ga 68 PSMA-11; Gallium Ga 68-labeled PSMA-11; Gallium Ga-labeled PSMA-11; Gallium-68 labeled PSMA-11; Gallium-68 PSMA; Gallium-68 PSMA Ligand Glu-urea-Lys(Ahx)-HBED-CC; Illuccix; ProstaMedix; PSMA-HBED-CC GA-68*  *Medical specialty: Oncology*  *Pharmacotherapeutic group: Diagnostic radiopharmaceuticals*  *Therapeutic area:* *Radionuclide Imaging*  *Orphan medicine: No*  *PRIME: priority medicines: No*  *Date of approval: 09.12.2022*  *ASMR rating: 5* | | | | |
| Advanced Accelerator Applications (AAA) | Acquired by Advance Accelarator Applications (AAA, 2010). AAA was acquired by Novartis for 3.9 billion USD in 2018. | YES | Basic, applied and translational research support:  Ga 68 PSMA-11 was co-developed by researchers at University of California, Los Angeles and University of California, San Francisco, who conducted a phase III clinical trial. | <https://shorturl.at/HYO0h>  <https://shorturl.at/yT7qn>  <https://shorturl.at/QCSoE> |
| Lutathera®  *Therapeutic agent: Lutetium Lu 177 dotatate*  *Alternative names: 77Lu-DOTA-TATE; 177Lu-DOTA0-Tyr3-Octreotate; 177Lu-labelled somatostatin analog peptide; [177Lu]Lu-DOTA-TATE; [Lu-177]-Dota-Tyr3-Octreotate; [Lu-177]-DOTATATE; AAA 601; F-1515; Lu-177-octreotate; Lu-DOTATATE; Lutate; LUTETIUM (177LU) OXODOTREOTIDE; Lutetium (177Lu) oxodotreotide - Advanced Accelerator Applications; Lutetium Lu 177 dotatate; lutetium Lu 177 dotatate; Lutetium-177-DOTA-Tyr-3-octreotate; Lutetium-177-DOTATATE; Octreotate Lu-177 DOTA Tyr-3; Octreotate-Lu-177*  *Medical specialty: Oncology*  *Pharmacotherapeutic group: Other therapeutic radiopharmaceuticals*  *Therapeutic area: Neuroendocrine Tumors*  *Orphan medicine: Yes*  *PRIME: priority medicines: No*  *Date of approval: 26.09.2017*  *ASMR rating: 3-5* | | | | |
| AAA | Acquired by AAA. AAA was acquired by Novartis for 3.9 billion USD in 2018. | YES | Basic, applied and translational research support:  Startup researchers developed the drug while being paid by University of Rotterdam.  Basic, applied and translational research support:  Phase 1 trial was carried out in Erasmus MC.  Technology transfer support to university spin-outs:  AAA is a spin-out from the European Organization for Nuclear Research (CERN). | <https://shorturl.at/yCjQT>  <https://shorturl.at/SsPxn>  <https://shorturl.at/VJDMs> |
| Luxturna®  *Therapeutic agent:* *Voretigene neparvovec*  *Alternative names: AAV2 hRPE65v2; AAV2-RPE65v2; Leber congenital amaurosis gene therapy; LTW 888;SPK-RPE65; voretigene neparvovec-rzyl*  *Medical specialty:* *Ophthalmology*  *Pharmacotherapeutic group: Other ophthalmologicals*  *Therapeutic area: Leber Congenital Amaurosis;Retinitis Pigmentosa*  *Orphan medicine: Yes*  *PRIME: priority medicines: No*  *Date of approval:* *22.11.2018*  *ASMR rating: 2* | | | | |
| The Childrens Hospital of Philadelphia (CHOP) | 2019: Spark Therapeutics => Spin-out of CHOP, bought by Roche  (Novartis owns ex-U.S. rights to Luxturna, while Roche holds U.S. rights through its acquisition of the therapy's developer, Spark Therapeutics | YES | Basic, applied and translational research support and  Technology transfer support to university spin-outs:  University of Pennsylvania and Children’s Hospital of Philadelphia | https://shorturl.at/q53zh  <https://shorturl.at/Lpq4y>  https://shorturl.at/qID6M  <https://shorturl.at/Yt3DJ> |
| Mayzent®  *Therapeutic agent:* *Siponimo*  *Alternative names: BAF-312; NVP BAF312 AEA; NVP-BAF312-NX; Siponimod fumarate*  *Medical specialty:* *Neurology*  *Pharmacotherapeutic group:* *Selective immunosuppressants*  *Therapeutic area: Multiple Sclerosis, Relapsing-Remitting*  *Orphan medicine: No*  *PRIME: priority medicines: No*  *Date of approval:* *13.01.2020*  *ASMR rating: n.a.* | | | | |
| Novartis | n.a. | n.a. | n.a. | n.a. |
| Mekinist®  *Therapeutic agent:* Trametinib  *Alternative names: 1120212; CE-Trametinib; GSK-1120212; GSK-1120212B; JTP-74057; Mecinist;Mekinisuto; Tasu Mekinisuto; TMT-212; Trametinib dimethyl sulfoxide; Trametinib DMSO*  *Medical specialty:* *Oncology*  *Pharmacotherapeutic group:* *Antineoplastic agents*  *Therapeutic area: Melanoma*  *Orphan medicine: No*  *PRIME: priority medicines: No*  *Date of approval:* *30.06.2014*  *ASMR rating: 5* | | | | |
| Japan Tobacco | 2015: As part of its purchase of oncology products from GlaxoSmithKline, Novartis obtained the worldwide exclusive rights granted by Japan Tobacco Inc. (JT) to develop, manufacture, and commercialize trametinib. JT retains co-promotion rights in Japan. | n.a. | n.a. | <https://shorturl.at/zQofD> |
| Piqray®  *Therapeutic agent:* *Alpelisib*  *Alternative names: Alpericive; BYL-719; NVP-BYL-719;Vijoice*  *Medical specialty:* *Oncology*  *Pharmacotherapeutic group:* *Antineoplastic agents*  *Therapeutic area: Breast Neoplasms*  *Orphan medicine: No*  *PRIME: priority medicines: No*  *Date of approval:* *27.07.2020*  *ASMR rating: n.a.* | | | | |
| Novartis | n.a. | n.a. | n.a. | n.a. |
| Pluvicto®  *Therapeutic agent:* *Lutetium-177 vipivotide tetraxetan*  *Alternative names: 177-Lutetium-PSMA-617 - Endocyte; 177Lu-EB-PSMA-617; 177Lu-PSMA; 177Lu-PSMA-617; 177LU-PSMA-617 - Endocyte; AAA617; Lu177 RLT; lutetium (177Lu) vipivotide tetraxetan; lutetium Lu 177 vipivotide tetraxetan - Advanced Accelerator Applications; Lutetium-177 PSMA 617*  *Medical specialty: Oncology*  *Pharmacotherapeutic group:* *Therapeutic radiopharmaceuticals*  *Therapeutic area: Prostatic Neoplasms, Castration-Resistant*  *Orphan medicine: No*  *PRIME: priority medicines: No*  *Date of approval:* 0*9.12.2022*  *ASMR rating: 3* | | | | |
| Endocyte; RadioMedix | 2018: Novartis successfully completes acquisition of Endocyte | YES | Basic, applied and translational research support and  Technology transfer support to university spin-outs:  University spin-out of Purdue University  Business support to SMEs & to innovative projects:  In 2001 Endocyte received 2 million USD from Indiana’s 21st Century Research and Technology Fund to continue clinical development of its oncology diagnostic and therapeutic products, purchase additional research equipment, and increase its research and development staff.  Basic, applied and translational research support:  Phase II clinical trial conducted by the Australian and New Zealand Urogenital and Prostate Cancer Trials Group (ANZUP) and the University of Sydney which jointly have the primary financial obligation and Endocyte providing financial support in exchange for access to data. | <https://shorturl.at/VjgvS>  <https://shorturl.at/V1R6n>  <https://shorturl.at/X5T84>  <https://shorturl.at/NOkWc> |
| Rydapt®  *Therapeutic agent:* *Midostaurin*  *Alternative names: 4-N-benzoyl staurosporine; Benzoylstaurosporine; CGP-41251; N-benzoyl-staurosporine; PKC-412; PKC-412A*  *Pharmacotherapeutic group:* *Antineoplastic agents*  *Medical specialty:* *Oncology*  *Therapeutic area: Leukemia, Myeloid, Acute;Mastocytosis*  *Orphan medicine: Yes*  *PRIME: priority medicines: No*  *Date of approval:* *18.09.2017*  *ASMR rating: 4-5* | | | | |
| Novartis | Collaboration between industry and academia. | YES | Basic, applied and translational research support:  Collaboration with different academic institutions. | <https://shorturl.at/EwhDS>  <https://shorturl.at/0MV9E> |
| Scemblix®  *Therapeutic agent:* *Asciminib*  *Alternative names: ABL 001; ABL-001 - Novartis; Asciminib hydrochloride - Novartis; Scemblix asciminib; STAMP inhibitor*  *Pharmacotherapeutic group: Antineoplastic agents*  *Medical specialty:* *Oncology*  *Therapeutic area: Leukemia, Myelogenous, Chronic, BCR-ABL Positive*  *Orphan medicine: Yes*  *PRIME: priority medicines: No*  *Date of approval:* *25.08.2022*  *ASMR rating: 4* | | | | |
| Novartis | n.a. | n.a. | n.a. |  |
| Simbrinza®  *Therapeutic agent:* *Brimonidine/brinzolamide*  *Alternative names: AL-4862/AL-8923A; AL-8923A/AL-4862; Brinzolamide/brimonidine*  *Pharmacotherapeutic group: Ophthalmologicals*  *Medical specialty:* *Ophthalmology*  *Therapeutic area: Ocular Hypertension;Glaucoma, Open-Angle*  *Orphan medicine: No*  *PRIME: priority medicines: No*  *Date of approval:* *18.07.2014*  *ASMR rating: 5* | | | | |
| Alcon | 2010: Acquisition of Alcon and then company spin-out from Novartis in 2019. | n.a. | n.a. | <https://shorturl.at/KYXQ3>  <https://shorturl.at/bM68A> |
| Tabrecta®  *Therapeutic agent:* *Capmatinib*  *Alternative names:* *INC-280; INCB-028060; INCB-28060*  *Medical specialty:* *Oncology*  *Pharmacotherapeutic group: Antineoplastic agents*  *Therapeutic area: Carcinoma, Non-Small-Cell Lung*  *Orphan medicine: No*  *PRIME: priority medicines: No*  *Date of approval:* *20.06.2022*  *ASMR rating: n.a.* | | | | |
| Incyte Corporation | 2009: MET inhibitor licensed to Novartis by Incyte Corporation. | n.a. | n.a. | <https://shorturl.at/7umyw> |
| Zolgensma*®*  *Therapeutic agent:* *Onasemnogene abeparvovec*  *Alternative names:* *AAV9-CBA-SMN1-gene-therapy-Novartis Gene Therapies; Adeno-associated-serotype-9-chicken-beta-actin-survival-motor-neuron-gene-therapy-Novartis Gene Therapies; AVXS 101; ChariSMA™; OAV-101; onasemno-gene abepar-vovec; onasemnogene abeparvovec-xioi; scAAV9.CB.SMN; SMA1-gene-therapy-Novartis Gene Therapies; SMN1-gene-therapy-Novartis Gene Therapies; SMNT-gene-therapy-Novartis Gene Therapies; Spinal-muscular-atrophy-gene-therapy-Novartis Gene Therapies; Survival-motor-neuron-1-gene-therapy-Novartis Gene Therapies; T-BCD541-gene-therapy-Novartis Gene Therapies; Telomeric-SMN-gene-therapy-Novartis Gene Therapies*  *Medical specialty: Neuromuscular*  *Pharmacotherapeutic group: Other drugs for disorders of the musculo-skeletal system*  *Therapeutic area: Muscular Atrophy, Spinal*  *Orphan medicine: Yes*  *PRIME: priority medicines: Yes*  *Date of approval:* *18.05.2020*  *ASMR rating: 3* | | | | |
| Nationwide Children's Hospital | 2018: Zolgensma was developed by AveXis which was acquired by Novartis (for detailed information about Zolgensma’s development history see Vokinger et al. (Vokinger et al., 2023)) | YES | Basic, applied and translational research support:  The University of Pennsylvania has highlighted James Wilson’s pivotal role in the development of the technology. According to the NIH RePORTER, James Wilson received over 35.8 million USD in funding from the NIH for research related to “adeno-associated virus” while at Penn. At NCH, researchers who worked on the development of Zolgensma included Brian Kaspar and Jerry Mendell, two scientists who have received more than 25 million USD in NIH grants, including millions for work on SMA.  Changes in ownership: licensing, acquisitions and merging:  AveXis licensed patents related to Zolgensma from Nationwide Children’s Hospital (NCH), the University of Pennsylvania, REGENX Biosciences (a firm created in 2009 by the University of Pennsylvania), and Genethon (a French charity). | <https://shorturl.at/hfRJs>  <https://shorturl.at/oYHQ5>  <https://shorturl.at/ik299> |
| Zykadia®  *Therapeutic agent:* *Ceritinib*  *Alternative names:* *Jikadia; LDK-378; NVP-LDK 378; NVP-LDK378-NX*  *Medical specialty:* *Oncology*  *Pharmacotherapeutic group: Antineoplastic agents*  *Therapeutic area: Carcinoma, Non-Small-Cell Lung*  *Orphan medicine: No*  *PRIME: priority medicines: No*  *Date of approval:* *06.05.2015*  *ASMR rating: 5* | | | | |
| Novartis | n.a. | n.a. | n.a. |  |

Supplement 2 Novo Nordisk' products that received EMA approval between 2014 and May 2024

| Origin company | Acquiring information | Public contribution | Type and categories of public funding | Source |
| --- | --- | --- | --- | --- |
| Esperoct®  *Therapeutic agent: Turoctocog alfa pegol*  *Alternative names: Long-acting recombinant factor VIII - Novo Nordisk; N8-GP; N8-GP rFVIII; NN 7170; NN-7088; NNC-0129-0000-1003; PEG turoctocog alfa; Pegylated turoctocog alfa; rFVIII glycopegylated*  *Medical specialty: Haematology*  *Pharmacotherapeutic group: Antihemorrhagics*  *Therapeutic area: Hemophilia A*  *Orphan medicine: No*  *PRIME: priority medicines: No*  *Date of approval: 20.06.2019*  *ASMR rating: n.a.* | | | | |
| Novo Nordisk | n.a. | n.a. | n.a. | n.a. |
| Fiasp®  *Therapeutic agent: Insulin aspart*  *Alternative names: Insulin aspart faster-acting; Faster aspartp; NN-1218*  *Medical specialty: Endocrinology*  *Pharmacotherapeutic group: Drugs used in diabetes, Insulins and analogues for injection, fast-acting*  *Therapeutic area: Diabetes Mellitus*  *Orphan medicine: No*  *PRIME: priority medicines: No*  *Date of approval: 09.01.2017*  *ASMR rating: 5* | | | | |
| Novo Nordisk (based on NovoLog which was developed by Novo Nordisk) | n.a. | n.a. | n.a. | <https://shorturl.at/ARgPu> |
| Ozempic®  *Therapeutic agent: Semaglutide*  *Alternative names: Semaglutide subcutaneous; 9931; NN-9535; NN-9536; NN-9931; NNC-0113-0217; Wegovy*  *Medical specialty: Endocrinology*  *Pharmacotherapeutic group: Drugs used in diabetes*  *Therapeutic area: Diabetes Mellitus*  *Orphan medicine: No*  *PRIME: priority medicines: No*  *Date of approval: 08.02.2018*  *ASMR rating: 5* | | | | |
| Novo Nordisk | n.a. | YES | Basic, applied and translational research support: controversy based on publically funded research on GLP-1.  Researchers Jens Juul Holst and Joel Habener received funding from Novo Nordisk on their GLP-1 research but did not patent their research on GLP-1. Basic research from Svetlana Mojsov played a critical role in the development on GLP-1 but she was not directly involved with the development of Semaglutide. | <https://shorturl.at/Aijhs>  <https://shorturl.at/KQ51L> |
| Refixia®  *Therapeutic agent:* *Nonacog beta pegol*  *Alternative names:* *onacog beta pegol; 40K PEG-rFIX; Glycopegylated rFIX - Novo Nordisk; La-rFIX derivative - Novo Nordisk; N9 GP; NN 7999; NNC-0156-0000-0009; REBINYN; Rebinyn*  *Medical specialty: Haematology*  *Pharmacotherapeutic group: Antihemorrhagics*  *Therapeutic area: Hemophilia B*  *Orphan medicine: No*  *PRIME: priority medicines: No*  *Date of approval: 02.06.2017*  *ASMR rating: n.a.* | | | | |
| Novo Nordisk | n.a. | n.a. | n.a. | n.a. |
| Saxenda®  *Therapeutic agent: Liraglutide*  *Alternative names: LATIN T1D; Liraglutide; NN 2211; NN 9211; NN-8022; NNC 90-1170;Victoza*  *Medical specialty: Endocrinology*  *Pharmacotherapeutic group: Drugs used in diabetes*  *Therapeutic area: Obesity;Overweight*  *Orphan medicine: No*  *PRIME: priority medicines: No*  *Date of approval: 23.03.2015*  *ASMR rating: n.a.* | | | | |
| Novo Nordisk | n.a. | YES | Basic, applied and translational research support: Collaboration with the University of Copenhagen | <https://shorturl.at/uODrm>  <https://shorturl.at/jPk8j> |
| Sogroya®  *Therapeutic agent: Somapacitan*  *Alternative names: Albumin-binding; Growth hormone derivative - Novo Nordisk; Long-acting growth hormone - Novo Nordisk; Long-acting somatropin - Novo Nordisk; NN 8640; NNC-0195-0092; Somatropin derivative - Novo Nordisk; Somatropin long acting - Novo Nordisk*  *Medical specialty: Endocrinology*  *Pharmacotherapeutic group: Pituitary and hypothalamic hormones and analogues*  *Therapeutic area: Growth*  *Orphan medicine: Yes*  *PRIME: priority medicines: No*  *Date of approval: 31.03.2021*  *ASMR rating: 5* | | | | |
| Novo Nordisk | n.a. | n.a. | n.a. | n.a. |
